# App-based support for parental self-efficacy in the first 1,000 days: A randomized control trial

**DOI:** 10.3389/fpsyg.2022.998170

**Published:** 2023-01-12

**Authors:** Laura A. Outhwaite

**Affiliations:** Centre for Education Policy and Equalising Opportunities, IOE, UCL’s Faculty of Education and Society, University College London, London, United Kingdom

**Keywords:** parental self-efficacy, randomized control trial, early child development, parenting app, first 1,000 days

## Abstract

Parental self-efficacy is key for guiding parents’ interactions with their child and is an important target for early intervention. This study reports a pilot randomized control trial (RCT) of a parenting application (app) with 79 parents of children aged 0–6 months in the United Kingdom. The app includes 1,026 daily age-appropriate activities across eight areas of child development, using resources accessible at home. While controlling for pre-test scores, parents who used the parenting app (Treatment Group) had significantly higher parental self-efficacy, after the 4-week intervention period, compared to the Active Control Group. Partial correlation analyses indicated that higher frequency of self-reported use of the parenting app was associated with greater parental self-efficacy outcomes. This evidence establishes proof of concept that parenting apps can have significant benefits on parental self-efficacy in early childhood. Limitations to the interpretation and generalization of the findings, as well as directions for future research are discussed.

## Introduction

The first 1,000 days of a child’s life, from conception to age 2, are an important period for child development (Wachs et al., 2015; Black et al., 2017). Evidence suggests healthy, secure, and playful social environments during this time play a role in promoting the acquisition of perceptual, motor, cognitive, language, socio-emotional, and self-regulation skills (Britto et al., 2017). These skills are important foundations for well-rounded child development and support later educational, physical, and mental health outcomes (Center on the Developing Child at Harvard University, 2009; Richter et al., 2021). For example, longitudinal cohort data from Germany, the Netherlands, and the United Kingdom (UK) indicates that variation in educational outcomes at age 11 can be significantly accounted for by home learning factors prior to the introduction of formal schooling (Passaretta et al., 2022).

While the first 1,000 days are important to child development, an overly deterministic focus on the very early years should be avoided (Macvarish et al., 2014). This is because it is not the only developmental period important for meaningful educational investment (Thompson and Nelson, 2001; Howard-Jones et al., 2012). Nevertheless, there are calls for quality interventions in the early years that supports parents to incorporate early learning opportunities in the home (Black et al., 2017; Eason et al., 2022). For example, interventions that can help parents to believe that their own actions and efforts can support children’s educational outcomes and improve the home learning environment in the early years (Goodman and Gregg, 2010). It is also recommended that nurturing care, including responsive caregiving and engagement in early learning from parents is a key component for effective intervention during the child’s first 1,000 days (World Health Organisation, 2020). Research suggests parental self-efficacy is an important target for these early interventions (Giallo et al., 2013), as it is a key mechanism guiding parents’ interactions with their child, including in the early home learning environment, which underpins later child outcomes (Albanese et al., 2019).

### Parental self-efficacy

Grounded in social cognitive and self-efficacy theory (Bandura, 1977), parental self-efficacy is defined as parents’ beliefs or judgments about their ability to be successful within their role as a parent (Eccles and Harold, 1996). Parental self-efficacy can be conceptualized as ‘task-specific,’ which focuses on parents’ beliefs or judgments about their ability to complete a specific task within a specific area of parenting (Leahy-Warren and McCarthy, 2011), such as feeding (Dennis and Faux, 1999). The current study adopted a ‘domain-specific’ approach to parental self-efficacy (Coleman and Karraker, 2000), which combines several of these ‘task-specific’ components to form a broad overview of parental self-efficacy (Barnes and Adamson-Macedo, 2007). Evidence suggests ‘domain-specific’ parental self-efficacy is a critical mechanism that guides parents’ interactions with their new-born child (Barnes and Adamson-Macedo, 2007) and is an important clinical target for the parent–child relationship, as well as parental mental health and later child development outcomes (Albanese et al., 2019), including school readiness (Patel and Corter, 2013).

In a recent systematic review, Albanese et al. (2019) found high parental self-efficacy was linked to positive parent–child relationships, including more responsive parenting practices (Montgomery, 2008), and increased attachment (Cassé et al., 2016). Further evidence suggests the relationship between parental self-efficacy and parenting competence is moderated by parental knowledge of child development, whereby mothers who reported high parental self-efficacy, but low knowledge of child development, were less sensitive in their play interactions with their young children, compared to mothers with high parental self-efficacy and high knowledge of child development (Hess et al., 2004). However, survey research in the UK indicates that only 11% of parents reported learning about child development prior to the birth of their first child (Beaver et al., 2020).

### Previous research

Previous intervention research shows in-person antenatal education programmes that focus on early parenting skills can have significant benefits for supporting the development of parental self-efficacy, compared to a business-as-usual control group (Svensson et al., 2009). Likewise, a recent systematic review of 102 experimental studies found that in-person parenting interventions implemented when children were aged 0–3-years led to significant increases in parenting knowledge and practice. Benefits were enhanced when programmes focused on increasing early play and learning opportunities (Jeong et al., 2021).

Further research has highlighted that digital delivery methods need to be considered to increase access to these supportive services for parents (Salonen et al., 2011). However, the current evidence base evaluating such approaches is fragmented and limited to a few studies. For example, a recent randomized control trial (RCT) found a web-based postnatal psychoeducational intervention, which focused on maternal and new-born care, as well as social support, had significantly stronger benefits on parental self-efficacy outcomes, compared to a business-as-usual control group and a home-based version of the same intervention (Jiao et al., 2019). Similarly, pilot studies have demonstrated the feasibility and acceptability of parenting applications (apps) for promoting parental self-efficacy in the context of postnatal maternal mental health (Chiou et al., 2021; Hsu et al., 2021). A systematic review and meta-analysis of seven studies also focused on postnatal maternal mental health found that digital or telephone-based parental interventions implemented for 4–17 weeks in the postpartum period had higher rates of completion, compared to business-as-usual postpartum care (Hanach et al., 2021).

Hanach et al. (2021) also descriptively found that digital-based parental interventions that were implemented for longer durations (measured in weeks) showed greater benefits on postpartum depression. In contrast, other systematic reviews of in-person parenting interventions found duration (measured in months) did not significantly impact parental outcomes (Jeong et al., 2021). However, in Jeong et al.’s (2021) review, intervention duration was dichotomously indicated as ‘more’ or ‘less’ than 12 months. This may have reduced the measurement sensitivity for detecting an association between frequency of activity use and intervention outcomes, and so could explain the conflicting findings. Despite the promising evidence for digital-based parenting interventions, no study to date has evaluated the impact of parenting apps on parental self-efficacy outcomes during a child’s first 1,000 days, and how frequency of activity use may be associated with the observed outcomes.

### Current study

To address this gap in the literature, the current study examined for the first time, the proof of concept of parenting apps with parents of children aged 0–6 months in the UK using a pilot RCT. The parenting app, at the focus of this study, is designed to support parents to engage and play with their child from conception to 2 years, as well as increase knowledge about their child’s development. Aligned with previous research (e.g., Jeong et al., 2021), these design features may provide support to facilitate early play and learning opportunities and thus led to improvements in parental self-efficacy.

As such, this study asked: do parents of children aged 0–6 months, have higher parental self-efficacy after using the parenting app (Treatment Group), compared to parents receiving similar activity ideas *via* email (Active Control Group)? Based on preliminary qualitative data (Outhwaite, 2020), it was predicted that parents who used the parenting app (Treatment Group) would have significantly higher parental self-efficacy than parents in the Active Control Group after the 4-week intervention period. This study also asked an exploratory research question: are increased parental self-efficacy outcomes associated with increased activity use of the parenting app (Treatment Group) and email activities (Active Control Group)?

## Materials and methods

### Design

A pilot RCT was conducted to establish proof of concept of a parenting app on parental self-efficacy, compared to an active control group (Green et al., 2019), with parents of children aged 0–6 months. This age range was chosen to minimize the potential heterogeneity of a sample across the full first 1,000 days. Eligible participants were randomly allocated to one of two parallel groups with a 1:1 ratio. The Treatment Group received the parenting app for 4 weeks. In comparison, the Active Control Group received a weekly email including three activity ideas per week for 4 weeks. All participants completed an external, validated assessment measure of parental self-efficacy at pre-test, before group allocation, and at post-test, immediately after the 4-week intervention period.

This RCT experimental design afforded the direct comparison between the target intervention, the parenting app, and an active control group. This enabled natural maturation and the effect of the intervention to be disentangled. Furthermore, the use of an active control group, whereby participants had access to alternative high-quality resources, was considered more ethical, in this context, compared to a business-as-usual/waiting list control group, where participants do not receive any support.

The UCL IOE Ethics Committee granted ethical approval for the study. Opt-in informed consent was obtained for all participants at the start of each online survey, in line with the British Psychological Society ethical guidelines and best practices in online research (Rodham and Gavin, 2006). 9.8% of participants who registered interest in the study agreed to take part. After the 4-week intervention period, all participants, regardless of group allocation, had continued access to the parenting app, free of charge.

### Sample size calculation

Guidelines from the Early Intervention Foundation’s (EIF) Levels of Evidence Framework recommend that pilot intervention trials (Level 2 preliminary evidence) have a minimum of 20 participants per group. A power calculation using the Optimal Design software (Raudenbush, 2011) showed in the context of this trial, an overall sample size of 40 participants (20 per group), with 80% power (*R*^2^ = 0.32, *p* = 0.05), the minimum detectable effect size (MDES) would be 0.76. The *R*^2^ value was estimated based on previous research on parental self-efficacy (e.g., Caldwell et al., 2011). Given the nature of this pilot study, the EIF recommendations for 20 participant per group was considered the minimum required sample size. To maximize the possibility of finding a medium effect size (0.50) and reduce the risk of falling below these recommendations due to potential attrition, a target sample size of 88 participants (44 per group) was established.

### Participants

**Figure 1:**
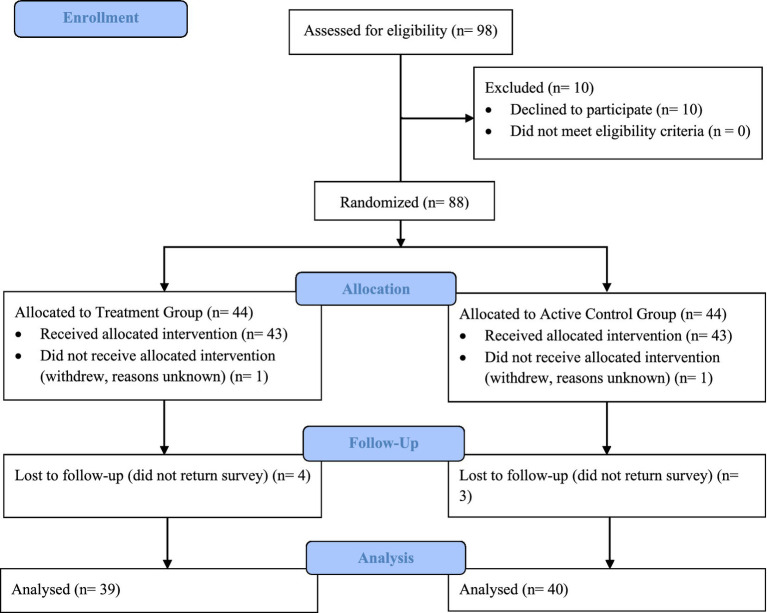
CONSORT (2010) flow diagram describing the composition of the study sample at each stage of the RCT.

Figure 1 summarizes the study sample at each stage of the RCT (CONSORT, 2010). In total, 98 potential participants were assessed for eligibility and invited to complete the pre-test survey. Eligibility criteria required participants to be aged 18 years or over and to be the parent or carer of a child aged 0–6 months. All potential participants met this eligibility criteria. Eighty-eight participants consented to take part in the study and returned their completed survey at pre-test. These participants were then randomly allocated to one of two groups. Forty-four participants were assigned to the Treatment Group and used the parenting app for 4 weeks. The remaining 44 participants were assigned to the Active Control Group and received a weekly email including three activity ideas per week for 4 weeks. Of these 88 participants, 79 completed the post-test survey at the end of the 4-week intervention period. In total, nine participants withdrew from the study (10.2% attrition rate), including seven participants who did not return follow up survey at post-test and two participants who withdrew from the study for reasons unknown. In line with ethical approval for this study, the data for these two participants were excluded from analysis.

Within the final sample of 86 enrolled participants, 93.0% reported living with their partner, the remaining 7.0% reported living with other adult family members or alone (including with other children). 87.2% of participants reported a higher education undergraduate or postgraduate degree as their highest level of education. The remaining 12.8% had further vocational training, school leaving qualifications, or no formal qualifications. 84.9% of participants identified as White British, the remaining 15.1% identified as Indian, Mixed, or Asian. The most common UK geographical regions represented in the sample were London (19.8%), the Southeast (24.4%), and the East of England (29.1%). The remaining 26.8% of participants were from other regions in England and Scotland. All parents reported feeling confident or very confident with technology. All babies were born during the COVID-19 pandemic, of which 95.3% were born full term and 4.7% were born pre-term before 37 weeks. Table 1 summarizes further descriptive statistics for the final sample of 86 participants.

**Table 1:** Descriptive data for the final sample in the trial (*n* = 86).

Descriptive data	Whole sample (*n* = 86)	Treatment group (*n* = 43)	Active control group (*n* = 43)
Parent age (years) Mean (SD), Min-Max	33.88 (4.05), 20.00–46.00	33.19 (3.94), 20.00–40.00	34.58 (4.08), 25.00–46.00
Baby age (weeks) Mean (SD), Min-Max	14.58 (7.24), 1.00–29.00	14.95 (7.20), 1.00–27.00	14.21 (7.33), 2.00–29.00
Gender of parent (F: M)	85: 1	42: 1	43: 0
Gender of baby (F: M)	46: 40	24: 19	22: 21
First time parent (Y: N)	57: 29	29: 14	28: 15
Attend antenatal classes (Y: N)	45: 41	19: 24	26: 17
EPDS score Mean (SD), Min-Max	8.76 (4.57), 0.00–23.00	8.74 (5.09), 1.00–23.00	8.77 (4.05), 0.00–20.00

EPDS, Edinburgh postnatal depression score (Cox et al., 1987).

### Treatment group

The intervention consisted of a parenting app known as Oliiki, developed by Clare Stead. The parenting app is designed to support parents to engage and play with their child from conception to 2 years. It includes 1,026 daily age-appropriate activities organized around eight areas of child development including, language, emotional control, habitual ways to respond, hearing, conceptualisation, vision, number, and peer social skills (Britto et al., 2017). Each activity explains to parents what to do and how to do it using resources easily accessible in the home. The app also explains to parents how activities are linked to child development by providing the research evidence behind the learning activities. Prior to birth, the parenting app focuses on helping users understand their role as a parent, and the impact they can have on their child’s development and education through communication with their partner or social support network. Once the child is born, the app provides play-based activities that support parent–child interactions.

**Figure 2:**
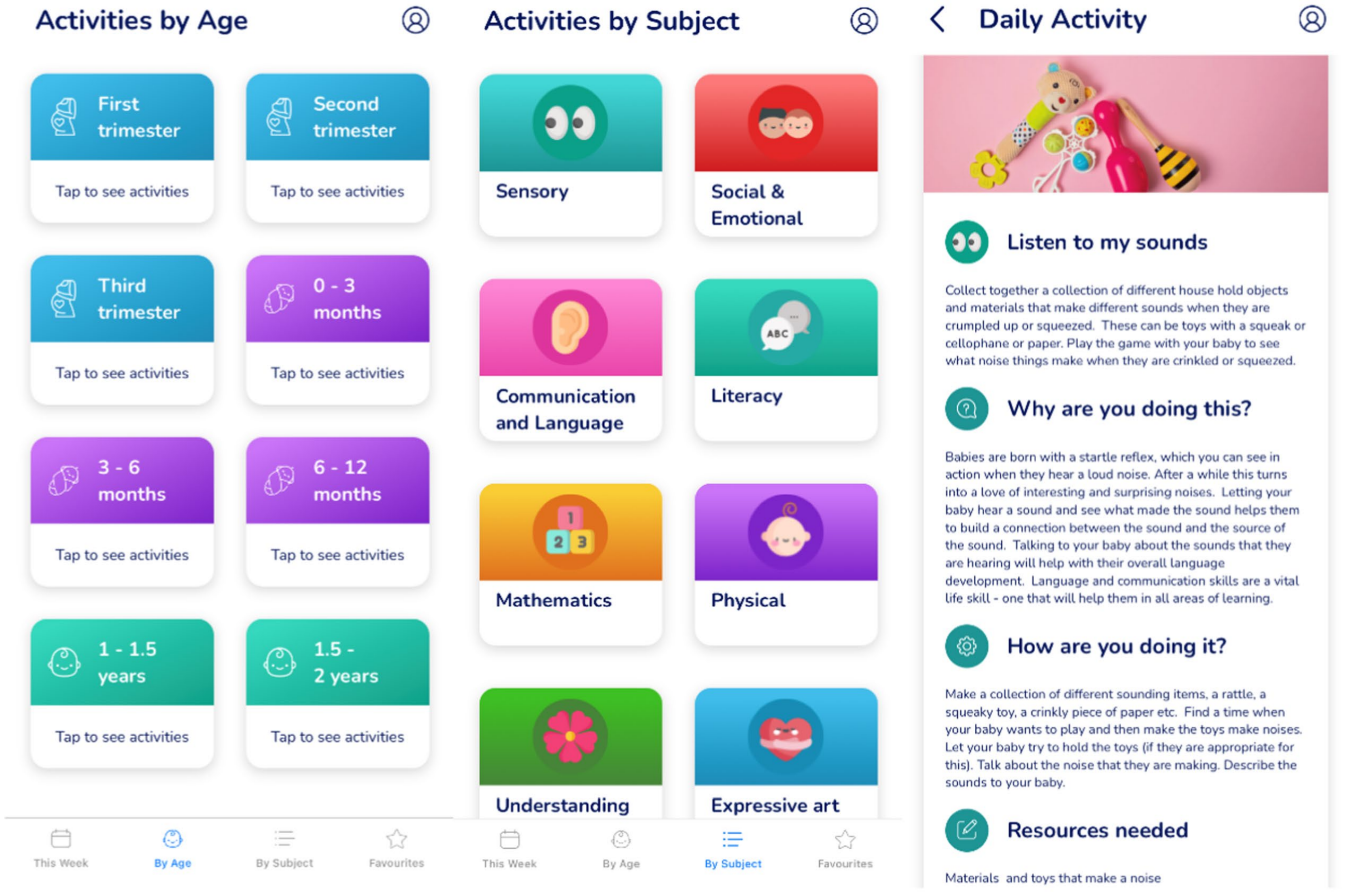
Example screenshots taken from the new parenting app intervention (courtesy of Oliiki).

For example, one activity designed to support children’s hearing focuses on playing with different everyday objects and materials that have different sounds. After describing the activity, the app provides a brief description of why these kinds of activities are important for child development. In this example, the app briefly describes how children are born with a startle reflex and it is important for them to make the connection between the sound and its source. The app then provides more details on how to implement the activity and the resources needed, followed by some of the underpinning research evidence. Overall, the app aims to build parent’s confidence and inspire ideas that can give all children the best start in life. Figure 2 includes example screenshots taken from the parenting app (courtesy of Oliiki).

### Active control group

During the 4-week intervention period, participants allocated to the Active Control Group were sent weekly emails containing three activity ideas. These activities were selected from the bank of activity ideas within the parenting app. However, the weekly emails only provided brief descriptions on what to do for each activity. No addition details were provided, and activities were not tailored to the child’s age or stage of development. Table 2 summaries the components of the parenting app (Treatment Group), in contrast to the weekly email activities (Active Control Group). Based on preliminary qualitative data (Outhwaite, 2020), it was predicted that the detail included within the parenting app, specifically the age-appropriate daily activities that parents could do with their child and explanations for why they are important, would be the active ingredients underpinning the hypothesized increase in parental self-efficacy.

**Table 2:** Comparison of intervention components for the parenting app (Treatment group) and the weekly email activities (Active control group).

Intervention components	Parenting app	Weekly email activities
Regular activities parents can do with their children	✓	✓
Ability to save favorite activities and revisit content	✓	
Activities are specifically designed for child’s age	✓	
Instructions on how to implement activities at home	✓	
Why the activities are important for child development	✓	
Explanations for research evidence underpinning the activities	✓	
Ability to track child’s progress in different areas of learning	✓	
Easy to access app technology format	✓	

### Parent self-efficacy outcome measure

Evidence suggests parental self-efficacy underpins several important factors in early child development and care, including the parent–child relationship, parental mental health, and later child development outcomes (Giallo et al., 2013; Albanese et al., 2019). As such, it was chosen as the primary, near-transfer variable.

Parental self-efficacy was measured using the Perceived Maternal Parental Self-Efficacy questionnaire (PMPSE; Barnes and Adamson-Macedo, 2007). This ‘domain-specific’ measure of parental self-efficacy measure was chosen based on its high-quality assessment score reported in Wittkowski et al. (2017). The PMPSE is a 20-item self-report questionnaire that measures maternal self-efficacy in the postpartum period across four factors including, Care taking procedures (e.g., “I am good at keeping my baby occupied”), Evoking behaviors (e.g., “I am good at getting my babies attention”), Reading behavior(s) or signaling (e.g., I can read my baby’s cues), and Situational beliefs (e.g., I believe that my baby and I have a good interaction with each other). Each item is rated on a 4-point Likert scale from ‘strongly disagree’ (scored 1) to ‘strongly agree’ (scored 4). Total raw scores range from 20 to 80, with higher scores indicating higher parental self-efficacy. The PMPSE has been used in similar intervention studies with parents of children aged 0–6 months with success (e.g., Jiao et al., 2019). The psychometric properties of PMPSE are rated highly (Wittkowski et al., 2017), with Cronbach alpha values in the region of 0.91–0.92 (Barnes and Adamson-Macedo, 2007). The Cronbach alpha value was 0.91 in this study.

### Procedure

#### Recruitment

Participants were recruited using opportunity sampling through social media advertisements. Participants already had access to a touch-screen tablet or mobile phone device required to download and use the parenting app. All participants were given access to the app, free of charge for the duration of, and following the completion of the study.

#### Implementation fidelity

Implementation fidelity was measured through frequency of activity use per week (Humphrey et al., 2016). During the online survey at post-test, participants were asked to indicate, on average, how many times per week they had used the parenting app or email activities. Due to the online nature of the study, this self-report approach was considered the most practical. Self-report measures of implementation fidelity are considered reliable when implementers, in this case the parents, fully understand the intervention delivery expectations (Humphrey et al., 2016). In the current study, this was achieved through regular email communication with all participants and the research team.

#### Group allocation

Participants were allocated to either the Treatment Group or Active Control Group after completing the pre-test survey. Given the uncertainty surrounding the sample size that could be achieved within this pilot trial, a blocked randomization procedure was implemented. This ensured there were as close to equal number of participants in each of the two groups, regardless of how many people would ultimately be recruited into the study (Efird, 2011). Each block contained two participants with exactly one participant allocated to each group. The fixed blocked randomization code was generated using the ‘ralloc’ package in Stata (Ryan, 2018). In total, 90 blocks were created (180 participants); a greater number than was necessary. This allowed for continuous enrolment, if interest in this study was beyond the target sample size of 88 participants. Due to the nature of the intervention for participants and implementation demands for the research team, it was not possible to blind the group allocations.

#### Data collection

Before (pre-test) and immediately after (post-test) the 4-week intervention period, participants completed the PMPSE items through an online survey delivered *via* Qualtrics. Demographic participant data was collected at pre-test only and reported activity use was collected at post-test. To ensure confidentiality participants completed the online survey using a self-generated unique identification number.

#### Intervention implementation

Participants allocated to the Treatment Group were advised to use the parenting app three or more times per week for 4 weeks, starting in October 2020. During this 4-week period, participants in the Treatment Group were sent two reminder emails to ensure engagement with the intervention. Participants allocated to the Active Control Group were sent weekly emails containing three activity ideas for the same 4-week period. Participants were encouraged to use these activities throughout each specific week.

## Results

### Preliminary analyses

Table 3 reports descriptive data on parental self-efficacy scores before (pre-test) and immediately after (post-test) the 4-week intervention period, as well as frequency of self-reported activity use per week for each group. A one-way analysis of variance (ANOVA) showed no significant differences in parental self-efficacy scores across the two groups at pre-test, *F*(1,84) = 0.02, *p* = 0.887. Further preliminary analyses comparing participant characteristics across the two groups (see Table 1), showed no significant differences in parent age, *t*(84) = 1.62, *p* = 0.110, child age, *t*(84) = 0.48, *p* = 0.636, or EPDS score, *t*(84) = 0.02, *p* = 0.981. There were also no observed group differences for first time parent status, χ(1) = 0.05, *p* = 0.820, or antenatal class attendance, χ(1) = 2.28, *p* = 0.131. Thus, as baseline equivalence was established within the RCT design, a one-way analysis of covariance (ANCOVA) was conducted. In the ANCOVA, differences in parental self-efficacy scores measured at post-test were compared across the Treatment and Active Control Groups, with pre-test parental self-efficacy scores entered as a covariate (Thomas and Zumbo, 2012).^1^

**Table 3:** Group mean (SD), minimum-maximum for PMPSE scores at pre-test and post-test, as well as frequency of self-reported activity use during 4-week intervention period.

Descriptive data	Treatment group (*n* = 39)	Active control group (*n* = 40)
Perceived maternal parental self-efficacy (PMPSE) scores (max. 80)		
Pre-test Mean (SD), Min-Max	62.51 (7.28), 45.00–78.00	62.30 (6.35), 52.00–77.00
Post-test Mean (SD), Min-Max	67.87 (6.24), 56.00–79.00	65.05 (6.95), 53.00–78.00
Frequency of self-reported activity use per week		
Mean (SD), Min-Max	3.95 (1.21), 1.00–6.00	2.50 (0.68), 1.00–3.00

### Parental-self efficacy

Results from the ANCOVA showed that while controlling for parental self-efficacy scores at pre-test, the Treatment Group had significantly higher parental self-efficacy scores at post-test, after using the parenting app for 4 weeks, compared to the Active Control Group, *F*(1,78) = 5.39, *p* = 0.023. This between-group difference at post-test was characterized by a Cohen’s *d* effect size of 0.43 (95% CI = −0.02–0.87).

### Frequency of activity use

Partial correlations, controlling for parental self-efficacy scores at pre-test, showed within the Treatment Group there was a positive and statistically significant relationship between parental self-efficacy scores at post-test and frequency of activity use across the 4-week intervention period, *r* = 0.39, *p* = 0.015. The same relationship was not observed for the Active Control Group, *r =* 0.02, *p* = 0.905. However, an independent samples t-test showed the frequency of activity use was significantly higher in the Treatment Group, compared to the Active Control Group, *t*(77) = 6.57, *p* < 0.001.

## Discussion

This study reports the first pilot RCT to establish proof of concept of the benefits of parenting apps on parental self-efficacy for parents of children aged 0–6 months. This study used online methods to work with participants during the COVID-19 pandemic in the UK: a time when parents of very young children were in most need (Best Beginnings et al., 2020). A ‘domain-specific’ approach to parental self-efficacy was adopted (Coleman and Karraker, 2000; Barnes and Adamson-Macedo, 2007), which encompasses parents’ beliefs and judgments in their ability to successfully engage with a range of behaviors associated with parenting, such as care taking procedures, evoking behaviors, reading behavior(s) or signaling, and situational beliefs (Barnes and Adamson-Macedo, 2007). The current findings are of particular significance for providing effective and accessible early learning interventions targeted at parents to support their children in the first 1,000 days (Beaver et al., 2020; Best Beginnings et al., 2020; World Health Organisation, 2020).

As predicted, result showed that while controlling for pre-test scores, parents who used the parenting app (Treatment Group) for 4 weeks had significantly higher parent self-efficacy, compared to those in the Active Control Group. Practically speaking, these results were characterized by a small effect size (between groups Cohen’s *d* = 0.43). Although, the scoring of the PMPSE measure does not equate to a threshold of ‘high’ or ‘low’ parental self-efficacy, evidence shows it can distinguish between respondents along a continuum of ‘higher’ and ‘lower’ parental self-efficacy. For example, parents who had previously given birth scored, on average, 4 points higher than first time mothers. This difference was found to be statistically significant. In contrast, parents of children born full-term scored, on average, 1.5 points higher than parents of pre-term children, and this difference was not found to be statistically significant (Barnes and Adamson-Macedo, 2007). In the current study, there was an average point score difference of 2.8, in favor of the Treatment Group at post-test. Furthermore, 89.7% of parents in the Treatment Group reporting feeling ‘confident’ or ‘very confident’ on all the PMPSE items after the 4-week intervention period, compared to 77.5% of parents in the Active Control Group.

Further exploratory partial correlation analyses also demonstrated that the higher frequency with which parents used the parenting app was significantly associated with greater parental self-efficacy outcomes (*r* = 0.39). The same relationship was not observed for the Active Control Group. However, this may be due to less overall frequency of activity use in the Active Control Group, compared to the Treatment Group (see Table 3). It is also important to caveat that within the context of the current pilot study, frequency of app and activity use by parents was indicated *via* self-report measures. Although this was considered most practical and has been shown to be reliable (Humphrey et al., 2016), it may be considered less objective compared to usage data collected automatically by the app. At the time of the current pilot study, this data was not available. Nevertheless, this evidence, indicatively suggests that the parenting app can have significant benefits on parental self-efficacy, even in a relatively short period of time: in this case 4 weeks. These findings are consistent with other studies of digital parenting interventions for mothers in the postnatal period (Jiao et al., 2019) and provide further support for digital intervention delivery methods (Hanach et al., 2021).

Within the current study, it should be noted that both the Treatment Group (within-group effect size, Cohen’s *d* = 0.79, 95% CI = 0.14–1.44, average 5.3% increase) and the Active Control Group (within-group effect size, Cohen’s *d* = 0.41, 95% CI = −0.21–1.04, average 3.4% increase) increased in parental self-efficacy over time. This may be, in part, due to natural maturation experienced by both groups over the 4-week intervention period. Some improvement in parental self-efficacy is to be expected as parents gain more experience with their new child. Furthermore, participants in both groups were given access to activity ideas. However, the observed gains in parental self-efficacy, across the four domain-specific factors (i.e., care taking procedures, evoking behaviors, reading behavior(s) or signaling, and situational beliefs), were greater for those in the Treatment Group. This suggests the active ingredients of the parenting app in comparison to the weekly activity emails, were understanding why the age-appropriate activities were important for child development with clear descriptions on how to implement the activities, combined with the research evidence underpinning the activity (see Table 2).

### Policy and practice implications

The current findings are of interest to policy and practice related to the need for effective and accessible interventions to support parents to engage with their child’s early learning and play in the first 1,000 days (Beaver et al., 2020; Best Beginnings et al., 2020; World Health Organisation, 2020). This pilot study has established proof of concept that parenting apps can support parental self-efficacy with parents of children aged 0–6 months. Furthermore, the current pilot study can be considered to have high internal validity and a low risk of bias through the successful randomization to group with baseline equivalence, the inclusion of an active control group, and the use of an external and validated assessment measure of parental self-efficacy, as well as an adequate and appropriately powered sample size with a relatively low level of attrition (10.2%). This means that the current findings can contribute to policy and practice discussions on this topic with confidence. However, there are three limitations to consider within this pilot study, which may affect the interpretation and generalization the current findings. These limitations can also help guide future research and scaling.

### Limitations and future directions

First, the current study was only implemented for 4 weeks. This decision was made based on a balance between practical constraints within the context of a pilot RCT and what was considered a minimum implementation period based on similar digital interventions studies previously conducted (Hanach et al., 2021). However, there is evidence to suggest that interventions with a shorter duration have a higher risk of inflated effect sizes, compared to interventions that are implemented for a longer duration, due to novelty effects (Cheung and Slavin, 2013). As part of a staged approach to scaling and to establish the efficacy of the parenting app (Green et al., 2019), future research should implement the intervention for a longer duration. Recommendations within educational research suggest a minimum intervention period of 12 weeks, as it enables the experience of the intervention to be well established (Higgins et al., 2012).

Second, the current study collected demographic and additional health information from the participants to understand the composition of the sample. Although there were no significant group differences in terms of parent age, child age, EPDS score, first time parent status, and antenatal class attendance, the current study sample was predominately White British, University-level educated, and living with a partner. This may limit the generalisability (external validity) of the current findings to other population groups. Further research should build on the current pilot RCT with more diverse population groups, particularly those in need of additional antenatal support. This will allow further investigation of who benefits the most from parenting apps and how it is most effectively implemented with the different groups (Pawson and Tilley, 1997). For example, based on theoretical models for the development of parental self-efficacy, the observed outcomes in the current study may be enhanced through the implementation of the parenting app alongside other social supports, such as partners, family, other mothers in similar circumstances, and trusted health and educational professionals (Leahy-Warren and McCarthy, 2011). Overall, this will help support policy and practice decisions on ensuring that all parents, particularly those most in need, have access to effective early learning interventions in the first 1,000 days.

Finally, the current study focused exclusively on parental self-efficacy, which in the context of this intervention may be considered a primary outcome or near-transfer variable (Green et al., 2019). Now that proof of concept has been established in this domain, further research is needed to examine the potential secondary or far-transfer benefits of the intervention. For example, there is a well-established link between parental self-efficacy, the parent–child relationship, parental mental health, and later child outcomes (Albanese et al., 2019). Future research examining these factors will benefit from the longer intervention duration and larger sample size already mentioned. Overall, future studies should also be pre-registered to ensure the confidence in results and subsequent policy and practice recommendations (Lochman, 2022).

## Conclusion

Overall, this study responds to the need for effective and accessible early learning interventions targeted at parents to support their children in the first 1,000 days (Beaver et al., 2020; Best Beginnings et al., 2020; World Health Organisation, 2020). The current findings demonstrate proof of concept that parenting apps can have significant benefits on parental self-efficacy. Furthermore, through its app-based method of delivery it can increase access for all parents, especially when postnatal services may be restricted during the COVID-19 pandemic. These findings have important implications for policy makers and practitioners seeking high-quality, accessible early learning interventions.

## Data availability statement

The raw data supporting the conclusions of this article will be made available by the authors, without undue reservation.

## Ethics statement

The studies involving human participants were reviewed and approved by the IOE-UCL’s Faculty of Education and Society. The patients/participants provided their written informed consent to participate in this study.

## Author contributions

LO: conceptualisation, methodology, formal analysis, writing–original draft, writing–review and editing, and project administration.

## Conflict of interest

The author declares that the research was conducted in the absence of any commercial or financial relationships that could be construed as a potential conflict of interest.

## Publisher’s note

All claims expressed in this article are solely those of the authors and do not necessarily represent those of their affiliated organizations, or those of the publisher, the editors and the reviewers. Any product that may be evaluated in this article, or claim that may be made by its manufacturer, is not guaranteed or endorsed by the publisher.

## Footnotes

^1^Although an ANCOVA analysis was deemed most appropriate within the context of the current study (Thomas and Zumbo, 2012), a One-way Analysis of Variance (ANOVA) using difference scores (post-test minus pre-test) was also conducted. The same pattern of results was observed, thus ensuring the robustness of the current findings.
